# Protocol registration improves reporting quality of systematic reviews in dentistry

**DOI:** 10.1186/s12874-020-00939-7

**Published:** 2020-03-11

**Authors:** Mateus Bertolini Fernandes dos Santos, Bernardo Antônio Agostini, Rafaela Bassani, Gabriel Kalil Rocha Pereira, Rafael Sarkis-Onofre

**Affiliations:** 1 Graduate Program in Dentistry, Federal University of Pelotas, 457 Gonçalves Chaves Street, Pelotas, 96015-560 Brazil; 2 Graduate Program in Dentistry, Meridional Faculty/IMED, 304 Senador Pinheiro Machado Street, Passo Fundo, 99070-220 Brazil; 3 The Bias, Reporting, Implementation, Guidance, ETHics, IntEgrity of and Reproducibility in Research (BRIGHTER) Meta Research Group, Porto Alegre, Brazil

**Keywords:** Systematic review, Protocol registration, Reporting, Outcome reporting bias, Dentistry

## Abstract

**Background:**

The aims of this study were to assess whether the previous registration of a systematic review (SR) is associated with the improvement of the quality of the report of SRs and whether SR registration reduced outcome reporting bias.

**Methods:**

We performed a search in PubMed for SRs in dentistry indexed in 2017. Data related to SR registration and reporting characteristics were extracted. We analyzed if the reporting of 21 characteristics of included SRs was associated with the prospective registration of protocols or reporting of a previously established protocol. The association between prospective registering of protocols, reporting of funding and number of included studies versus outcome reporting bias was tested via multivariable logistic regression.

**Results:**

We included 495 SRs. One hundred and 62 (32.7%) SRs reported registering the SR protocol or working from a previously established protocol. Thirteen reporting characteristics were described statistically significant in SRs registered versus SRs that were not. Publication bias assessment and Report the number of participants showed the highest effects favoring the register (RR 1.59, CI 95% 1.19–2.12; RR 1.58, CI 95% 1.31–1.92 respectively). Moreover, Registration was not significantly linked with the articles’ reporting statistical significance (OR 0.96, CI 95% 0.49–1.90).

**Conclusion:**

There is a positive influence of previously registering a protocol in the final report quality of SRs in dentistry. However, we did not observe an association between protocol registration and reduction in outcome reporting bias.

## Background

Systematic reviews (SRs) are an important tool for health professionals that help them during decision-making processes to define a diagnostic, prevention and/or treatment of a disease, disorder and/or condition [1, 2]. Although SRs are recommended to summarize the present evidence in a specific field of knowledge, they are dependent on the availability, quality and assessed outcomes of the included studies. In addition, the conducting of a SR could affect the obtained results, thus a rigorous and replicable methodology is mandatory [1, 3].

Over the last years, a considerable increase in SRs has been noticed [4], including in dentistry [5, 6]. However, Page et al. [4] along with Ioannidis (2016) [7] observed that the increase of such publications was accompanied by an increase of poorly conducted, reported and/or unnecessary SRs. In this way, certain initiatives were initiated to reduce bias in the conduct and reporting of SRs. The International Prospective Register Of Systematic Reviews (PROSPERO) initiative is an international database of prospectively registered SRs, where protocols for SRs should be submitted containing key information about the design and conduct of the SR prior to its start [8]. The advantages of registering SR protocols could be related to preventing duplication of SRs and assessment of any outcome reporting bias, which can happen when the reported outcome is selected a posteriori, based on the results. The presence of that bias could be assess comparing the protocol and final manuscript [8, 9].

The prospective register of SR requires the development of study protocol as a road map of study development and to develop that protocol reviewers should be familiar with the PRISMA Statement and the requirements of PROSPERO. Consequently, the prospective register and development of study protocol could be a strategy to improve the methodological and reporting quality. A recent study observed that a priori registration of SR protocols in PROSPERO is associated with high methodological quality of orthodontics SRs [10]. Ge et al. (2018) [11] demonstrated that the prospective registration of SR protocols could be associated with the improvement of the overall methodological quality of a SR, however considering the reporting of the final manuscript, only a few items were better reported in registered SRs versus non-registered ones. In dentistry, there is a lack of evidence surrounding the association of prospective registration of SR protocols and reporting quality of final manuscripts. Thus, the goal of this study was to assess whether the previous registration of a SR or development of a protocol are connected with the improvement of the quality of the report of SRs. Additionally, we tested whether registration reduced outcome reporting bias.

## Methods

We used a database of SRs previously assembled consisting of SRs in dentistry indexed in PubMed in 2017. Characteristics of these SRs were previously published [5]. This is a meta-research study that followed the 4-phase flow set forth in the Preferred Reporting Items for Systematic Reviews and Meta-Analyses Statement [12] and compared the completeness of 21 reporting characteristics of SRs registered/reporting established protocols and non-registered SRs. The study selection and data extraction were conducted between 2018 January 20 until July 20.

### Search and eligibility criteria

A full description of the search strategy and eligibility criteria is available in the study of Bassani et al. 2019 [5]. Briefly, the search was conducted only in PubMed where dentistry-related SRs that assessed diagnosis, prevention and/or treatment of diseases, disorders and/or conditions of the oral cavity, maxillofacial and/or adjacent area and associated structures independent of the type of study indexed in 2017 (from January 01 until December 31) and published in the English language. The search strategy was based on MeSH terms of PubMed and a specific filter (U.S. National Library of Medicine) to retrieve reports of SRs. The full search strategy is presented in Supplemental Material. Articles reported as narrative/non-systematic literature reviews, rapid reviews, overviews of reviews (or umbrella reviews), scoping reviews, methodology articles evaluating quality of studies, comments and protocols or summaries of SRs and those published in languages other than English were excluded.

### Screening

Study selection was with reference manager software (EndNote X7, Thomson Reuters, New York, USA). Initially, we randomly selected 20 references to perform a pilot test screen to ensure consistency between the two reviewers involved during that phase using Microsoft Excel (Redmond, USA). Subsequently, two researchers (RB and RSO) identified, independently, articles by reviewing titles and abstracts for relevance. The retrieved records were classified as include, exclude or uncertain. The full-text articles of the included and uncertain records were selected for further eligibility screening by the same two reviewers. Discrepancies in screening of titles/abstracts and full-text articles were resolved through discussion. In the case of disagreement, the opinion of a third reviewer was garnered.

### Data extraction

A standardized score sheet was created using Microsoft Excel based on the data extraction form developed by Page et al. [4]. To ensure consistency, a pilot data extraction on a random sample of 10 included SRs was conducted. This pilot data extraction was carried out through a discussion between the reviewers (RB, GKRP, RSO) in order to consider all data for extraction. Subsequently, data from each SR were extracted by one of three reviewers (RB, GKRP, RSO). Data regarding category of the journal (general or specialty journal), number of studies per journal, number of authors, number of databases used in the SR search and SR focus (Epidemiology, Diagnosis, Prevention, Prognosis, Treatment/Therapeutic, Other, Unclear, or Mixed), dental specialty, funding (reported or not reported) and reported statistical significance of the first outcome were collected. After, one author verified the consistency of data and in the case of doubt or inconsistency, the data were extracted again.

To assess SR registration, five questions were posed: 1) Did the authors report registering the review or report working from a protocol?; 2) What is the name of the register? (PROSPERO, Joanna Brings, Other or Not reported); 3) Is a registration number provided? (Yes or No); 4) Is a hyperlink to the full registration record provided? (Yes or No); and 5) Where is the registration information shown in the report? (Abstract or Methods). With regard to the assessment of quality of the report, 21 reporting characteristics (Table 1) were evaluated for each included SR. All these domains are based on the PRISMA Statement and were selected because they are categorized dichotomously as “reported” and “not reported”.

**Table 1:** Quality of report assessment items

#	Assessed characteristics
1	SR or meta-analysis in title/abstract
2	Eligible publication status reported
3	Eligible languages reported
4	Eligible study designs reported
5	Both start and end years of search reported
6	Full Boolean search strategy reported
7	Screening method reported
8	Data extraction method reported
9	Risk of bias/quality of studies assessed
10	Risk of bias/quality assessment method reported
11	Review flow fully reported
12	Excluded studies fully reported
13	Total number of participants reported
14	Outcomes specified in Methods section
15	Primary outcomes specified
16	Statistical heterogeneity assessed
17	Publication bias assessed (or intent to assess)
18	Harms assessed (or intent to assess)
19	Both SR and study limitations reported
20	Abstract conclusions incorporate limitations
21	Source of funding of SR reported

We considered the primary outcome the completeness of 21 reporting characteristics and the secondary outcome the reporting of statistical significance of the first outcome.

### Data analysis

Descriptive analysis of the data was performed with data summarized as frequency for categorical items or median and interquartile range for continuous data using Stata 14.2 software. Characteristics of the SRs were assessed considering all SRs included, the reporting characteristics of the registered SRs and the number of non-registered and registered SRs by dental specialties.

We further analyzed if the reporting of 21 characteristics of the included SRs was associated with the prospective registration of the protocol or reporting of an established protocol. The proportion of SRs with adequate reporting of these items was calculated. Employing these proportions, we compared the completeness of reporting between SRs registered/reporting established protocols versus non-registered SRs calculating the Risk Relative (RR) with a 95% confidence interval for each characteristic. The analysis was performed in Review Manager (RevMan Copenhagen: The Nordic Cochrane Centre, The Cochrane Collaboration, 2014). As a sensitive analysis, we assessed these 21 characteristics comparing only SRs registered versus the rest of SRs.

Additionally, we used the reporting of statistical significance of the first outcome as a surrogate of outcome reporting bias based on a previous study [13]. Outcome reporting bias is the selective reporting of pre-specified outcomes in published studies and in cases of outcome reporting bias we can observe increase risk of reporting statistical significance. We used that considering the protocol registration could prevents discrepancies in the outcomes between protocols and the final manuscript and consequently the likelihood of reporting statistical significance in registered SRs would be reduced.

The first result could be identified from the abstract or results section of the review, depending on where it is first reported in the publication. The association between prospective registration of a protocol or reporting of an established protocol, reporting of funding and number of included studies along with outcome reporting bias was tested via multivariable logistic regression. The analyses were performed using Stata 14.2 and *P* ≤ 0.05 was regarded as statistically significant.

## Results

**Fig. 1:**
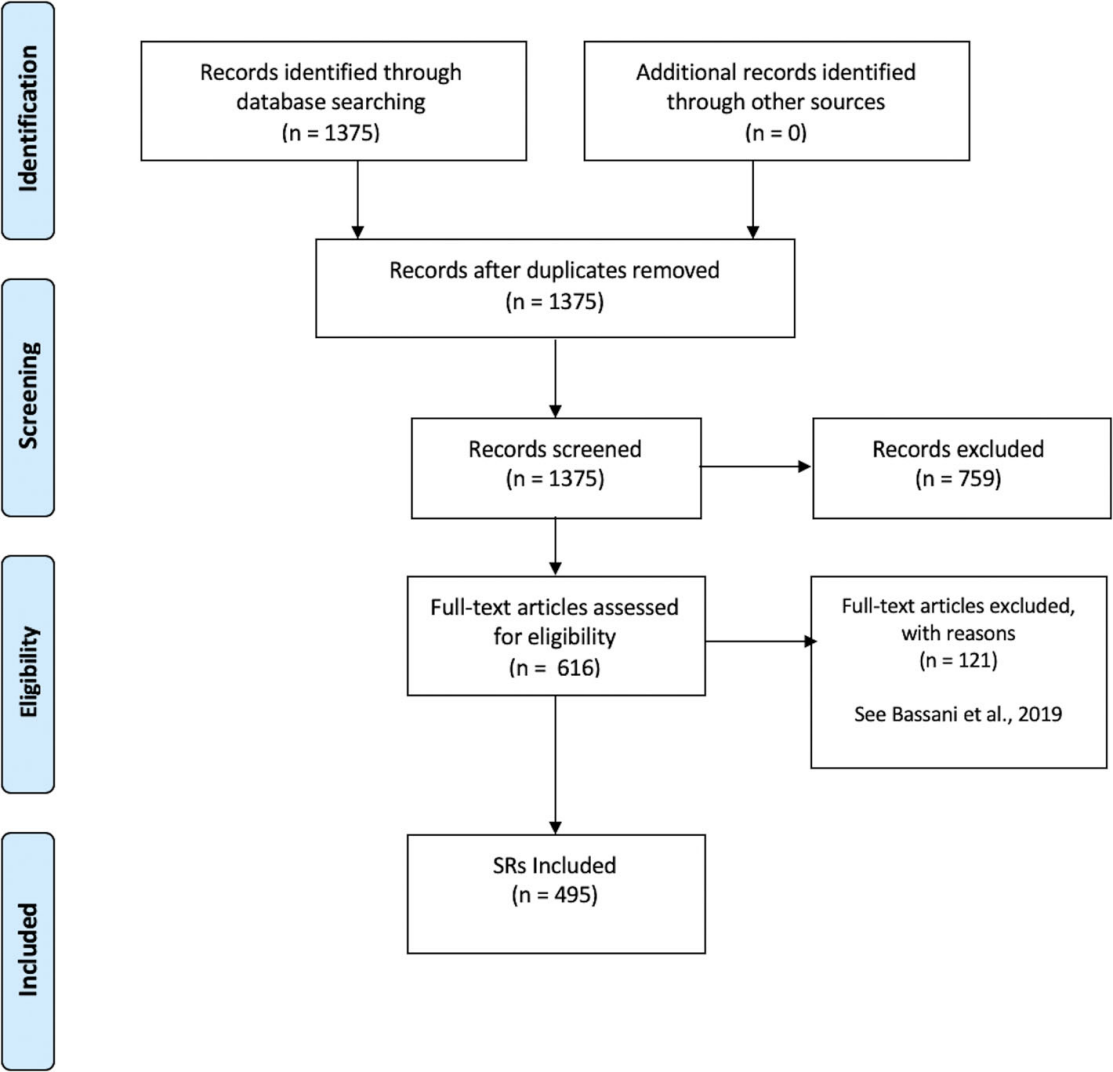
Flow diagram outlining study selection process

Figure 1 depicts a flow diagram outlining the review process. The initial search in PubMed yielded 1375 record and after study screening of the title/abstract and full-text analysis, we included 495 SRs (see Bassani et al., 2019).

Table 2 features the characteristics of the included SRs. One hundred and 49 (30.1%) SRs reported registered the SR protocol, 13 SRs (2.6%) reported working from a previously established protocol and 333 (67.3%) reported not registering the SR protocol or did not work from a protocol established earlier. Considering non-registered/no protocol SRs, most were published in a specialty journal (*n* = 242, 72.7%) and the main focus was treatment/therapy (*n* = 146, 43.8%) followed by diagnosis (*n* = 61, 18.3%). The same characteristic was noticed when considering registered/protocol SRs, whereas most were also published in a specialty journal (*n* = 120, 74.1%) and the main focus was treatment/therapy (*n* = 69, 42.6%) followed by diagnosis (*n* = 32, 19.8%). Besides this, registered, SR working from a previously established protocol and not registered SRs presented similar characteristics related to number of authors, included studies and databases searched.

**Table 2:** Characteristics of included SRs

Characteristic	Non-registered/no protocol (***n*** = 333)		Registered (*n* = 149)		Protocol *n* = 13	
	n	%	n	%	n	%
**Category of journal**						
General	91	27.3%	40	26.8%	2	15.4%
Specialty	242	72.7%	109	73.2%	11	84.6%
**SR Focus**						
Treatment/Therapeutic	146	43.8%	59	39.6%	10	76.9%
Diagnosis	61	18.3%	31	20.8%	1	7.7%
Prognosis	35	10.5%	17	11.4%	0	0%
Other	35	10.5%	10	6.7%	1	7.7%
Epidemiology	28	8.4%	16	10.7%	0	0
Unclear	11	3.3%	9	6.0%	0	0
Prevention	12	3.6%	6	4%	1	7.7%
Mixed	5	1.5%	1	0.7%	0	0
**Number of authors: median (IQR)**	5 (3–6)			5 (3–5)	5 (3–6)	
**Number of studies: median (IQR)**	14 (9–26)			14 (6–24)	16 (8–23)	
**Number of databases: median (IQR)**	3 (4–2)			4 (5–3)	3 (4–2)	

Considering only the SRs that reported the prospective registration of SR (*n* = 149), most were registered in PROSPERO (*n* = 135, 90.6%) and most reported the registration number (*n* = 134, 89.9%). Despite that, most SRs did not provide the hyperlink to the full registration record (*n* = 114, 76.5%). Finally, in most SRs, the registration information was listed in the Methods section (*n* = 144, 96.6%) (Table 3).

**Table 3:** Reporting characteristics of registered SRs

Characteristic	Number	Percent
**What is the name of the register?**		
PROSPERO	135	90.6%
Joanna Brings	5	3.4%
Other	4	2.7%
Not reported	5	3.4%
**Is a registration number provided?**		
Yes	134	89.9%
No	15	10.1%
**Is a hyperlink to the full registration record provided?**		
Yes	35	23.5%
No	114	76.5%
**Where is the registration information listed in the report?**		
Abstract	5	3.4%
Methods	144	96.6%

**Fig. 2:**
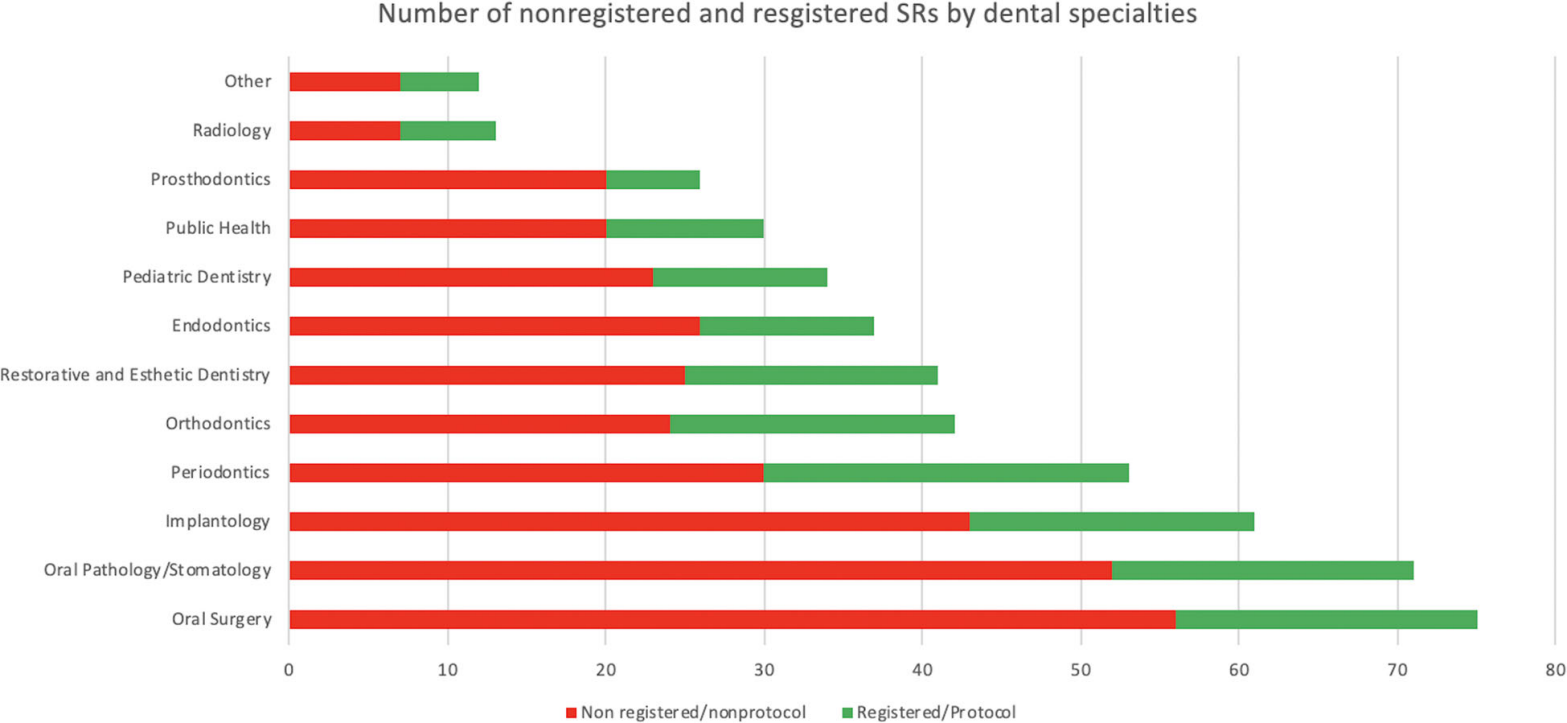
Number of non-registered and registered SRs by dental specialty

**Fig. 3:**
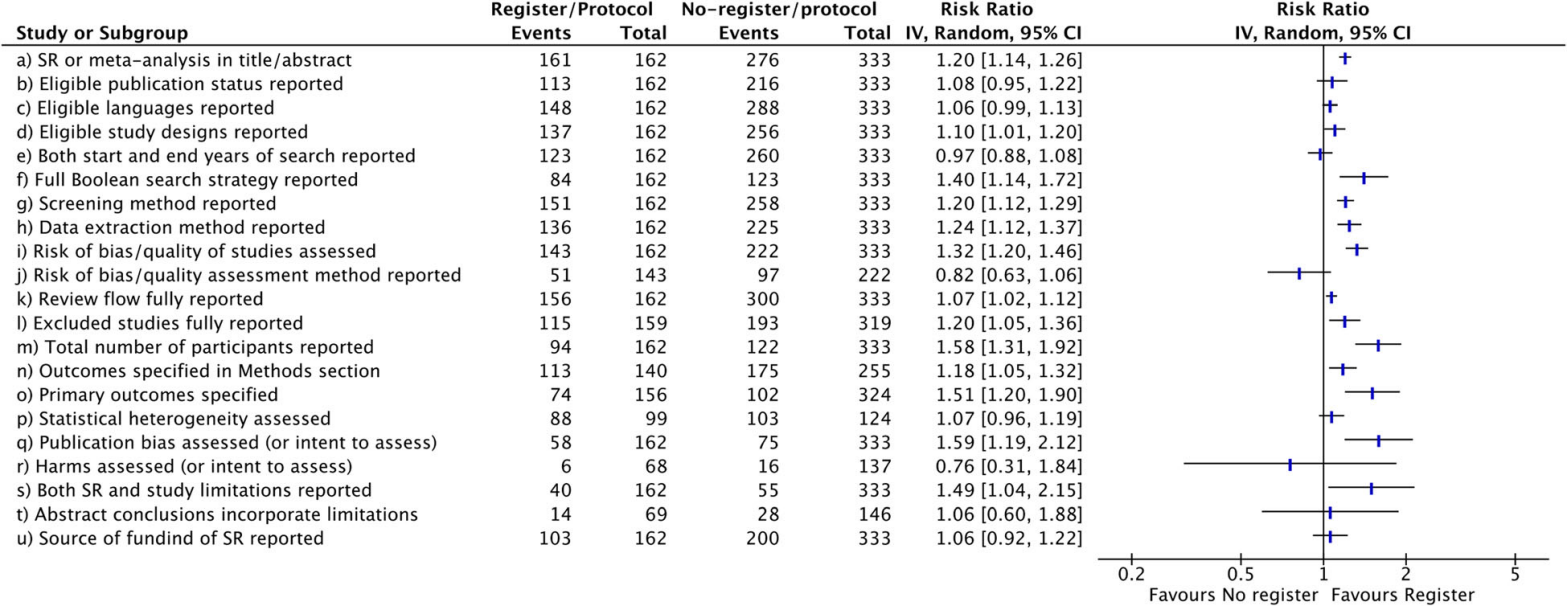
Pooled relative risks across assessed reporting characteristics of SRs with 95% confidence intervals comparing the completeness of reporting between SRs that are registered/reporting previously established protocols versus non-registered SRs

Figure 2 presents the number of non-registered and registered SRs by dental specialties and we can observe that all dental specialties presented a higher number of non-registered SRs than registered SRs. Figure 3 portrays the association between prospective registration of protocols and reporting characteristics. Thirteen reporting characteristics were described statistically significantly better in SR registered versus SRs that were not (SR or meta-analysis in title/abstract, Eligible study designs reported, Full Boolean search strategy reported, Screening method reported, Data extraction method reported, Risk of bias/quality of studies assessed, Review flow fully reported, Excluded studies fully reported, Total number of participants reported, Outcomes specified in Methods section, Primary outcomes specified, Publication bias assessed (or intent to assess), Both SR and Study limitations reported). The sensitive analysis demonstrated that 11 reporting characteristics were described statistically significantly better in SR registered versus SRs that were not (SR or meta-analysis in title/abstract, Full Boolean search strategy reported, Screening method reported, Data extraction method reported, Risk of bias/quality of studies assessed, Review flow fully reported, Excluded studies fully reported, Total number of participants reported, Outcomes specified in Methods section, Primary outcomes specified, Publication bias assessed (or intent to assess)). The figure of analysis is presented in the Supplementary material.

Table 4 presents the relationship between prospective registration of a protocol or reporting of an established protocol and variables of interest. Registration was not significantly associated with the reporting of statistical significance (OR 0.96 CI 95% 0.49–1.90).

**Table 4:** Analysis for reporting statistical significance

	OR	95%CI	*P*-value
**Register or protocol**			0.919
Presence	Ref.		
Absence	0.96	0.49–1.90	
**Funding**			0.053
Reported	Ref.		
Not Reported	0.48	0.23–1.01	
**n Studies Included**			0.342
0–13	Ref.		
≥ 14	1.39	0.70–2.73	

*OR* odds ratio, *CI* confidence interval, *Ref* reference

## Discussion

Our study is the first in the oral health literature to analyze whether the previous registration of a SR or development of a protocol is associated with the improvement of the quality of the reporting of SRs considering all dental specialties. In addition, few reports have assessed that association in the biomedical field [10, 11, 13], demonstrating there is a lack of evidence regarding this. In our results, approximately 60% of items evaluated (13 of 21 items) showed statistical association. All of those association favors the SRs registration over SRs that did not, it could indicate evidence surrounding the impact of registration protocols in the quality of reporting SRs in dentistry.

The aspects that showed a positive association with protocol registration were related to all sections of the SRs and it is important to highlight the absence of these aspects could lead to controversial or biased information. Moreover, poor or incomplete reporting could result in major problems: 1) invalidation of produced evidence; 2) irreproducible research; 3) questionable validity of the results; 4) unusable results; and 5) waste of resources [14]. The influence of such problems could be even greater in cases that consistency of the results determines the adoption of certain preventive and clinical practices (i.e., when the study establishes a causal relation between health outcome and risk factor). Studies with some sort of protocol determines standards that guide the report and prevents misunderstandings in study methods and, consequently, the validity of the results [10, 11].

Ge et al. (2018) assessed the association of prospective registration and improvement on reporting and methodological quality of SRs. The authors pointed out that the prospective registration could be associated with improvement in methodological quality, however the findings did not demonstrate association with improvement in reporting quality [11]. Additionally, Sideri et al. (2018) assessed a sample of orthodontics SRs and demonstrated that a small proportion of SRs were registered in PROSPERO, however registered SRs had higher methodological quality than non-registered SRs [10]. Our study strengthened these previous findings and further bolstered evidence that protocol registration significantly impacts the reporting quality of SRs.

We also assessed the influence of protocols in outcome reporting bias. In SRs, outcome reporting bias occurs when the decision of which outcome will be investigated is based on the results for those outcomes in the included studies or when none of the included studies assesses the outcome of interest [15]. Kirkham et al. (2010) suggested that statistically significant outcomes are more likely to be promoted from secondary to primary outcomes or included in final publications compared to the protocol [16]. Protocol registration avoids discrepancies in the assessment of outcomes between protocol and study development, ensuring transparency and reducing variability outcome specification [17]. However, the findings of Tricco et al. (2016) has demonstrated that many SRs registered in PROSPERO did not specify the primary outcome or changed it [18]. Our results did not find an association between protocol registration and outcome reporting bias. Tsujimoto et al. (2017) assessed 284 SRs published in high impact journals and demonstrated that protocol registration did not reduce outcome reporting bias and highlighted the need to compare the final manuscript and the protocol to detect reporting bias [13].

The majority (67.3%) of the SRs included did not register the protocol, however this is not a problem confined to dentistry. Tsujimoto et al. (2017) evaluated 284 SRs published in general and internal medical journals with the highest impact factors in 2013, showing that registration protocol reporting increased over earlier years, but the proportion is still low [13]. In contrast, Page et al. (2018) demonstrated that 30,000 SRs were registered in PROSPERO with a 10-fold increase in registrations comparing 2012 to 2017 [19]. The first negative impact related to non-adherence of protocol registration is the possibility of duplication of research questions, an aspect being one of the first aims of a protocol registration that was endorsed before [17]. Moreover, a number of positive aspects could be related to protocol registration: 1) provides the reader with an important tool to prevent outcome reporting bias and, consequently, publication bias; 2) could improve indirectly the overall methodological quality of the SR.

An important element to highlight is that PROSPERO presents specific inclusion criteria to register SRs. In dentistry, many SRs are based on in vitro studies and these SRs are not eligible for registration, and for this reason, we included in our analysis SRs only reporting working from a protocol. Thus, we suggest that PROSPERO has broader inclusion criteria and dental journals accept SR protocols for publication following the examples of biomedical journals that include *Systematic Reviews* and *BMJ Open*. Also, the use of open platforms such as OSF (https://osf.io) should be encouraged.

There are several limitations of our study. First, we only employed one database that featured articles published in English and considering only 1 year (2017), so the results may not be generalized to other databases and languages. Second, we did not distinguish the design of each SR. PROSPERO and the PRISMA statement [12] were firstly developed with respect to SRs of randomized controlled trials and could have overestimation of certain associations because adherence to various report items could not be applied. Third, some items of PRISMA Statement which could distort results, such as the assessment of the strength of the evidence for main outcomes and their relevance, methods for handling meta-analysis, were not assessed. Fifth, we conducted just unadjusted statistical analysis (did not include possible confounders) and did not measure possible interactions, this fact not allow the proper discuss of which are the critical methodological aspect influencing report. However, the aim of the paper was to explore possible influencers or indicators of quality report and was not an attempt to indicate the real effect of each characteristics in the final paper quality. Finally, we utilized a surrogate outcome to measure outcome reporting bias, and this does not directly reflect outcome reporting bias, however it could be a useful tool to identify it - outcomes were chosen based on statistical significance.

Our results indicated a positive influence of registration protocol in the report quality of SRs in dentistry, however we believe that there is room for improvement in the performance, reporting and prospective registration of SRs in dentistry. The following are certain suggestions for enhancement:

1. Researchers should be trained in all aspects of SRs; and
2. Dental journals, funding agencies, governments and universities should encourage the prospective registration of SRs as an important indicator of transparency in research.

In terms of clinical significance, the poor reporting of SRs could have serious consequences for health-care decision-making and wasting of time and resources. Problems with incomplete reporting could be a barrier to clinicians based on their decisions with respect to scientific reports and incorporating evidence-based medicine into practice. It seems that there is a positive influence of registering protocols in report quality of SRs and better reporting could lead to better decision-making for clinicians. Studies exploring a large number of reporting domains are still necessary.

## Conclusions

There is a positive influence of registering protocols on the report quality of SRs in dentistry. However, we did not observe an association between protocol registration and reduction in outcome reporting bias. Considering that registration or the development of a public protocol reduces waste of science, we endorsed the policy of using registration protocols as mandatory for further SRs in dentistry, thereby producing more reliable and transparent evidence.

## Supplementary information

**Additional file 1.** Study or Subgroup.

**Additional file 2.** Reporting criteria.

**Additional file 3.** Rating of each SR considering 21 reporting characteristics.

## Data Availability

All data generated or analysed during this study are included in this published article.

## Abbreviations

OR: Odds Ratio
PRISMA: Preferred Reporting Items for Systematic Reviews and Meta-Analyses Statement
PROSPERO: International Prospective Register Of Systematic Reviews
SR: Systematic review
