# Supplementary figures and images for: Protocol registration improves reporting quality of systematic reviews in dentistry
Source: BMC Med Res Methodol. 2020 Mar 11;20:57. doi: 10.1186/s12874-020-00939-7 (PMC7065343; doi:10.1186/s12874-020-00939-7)

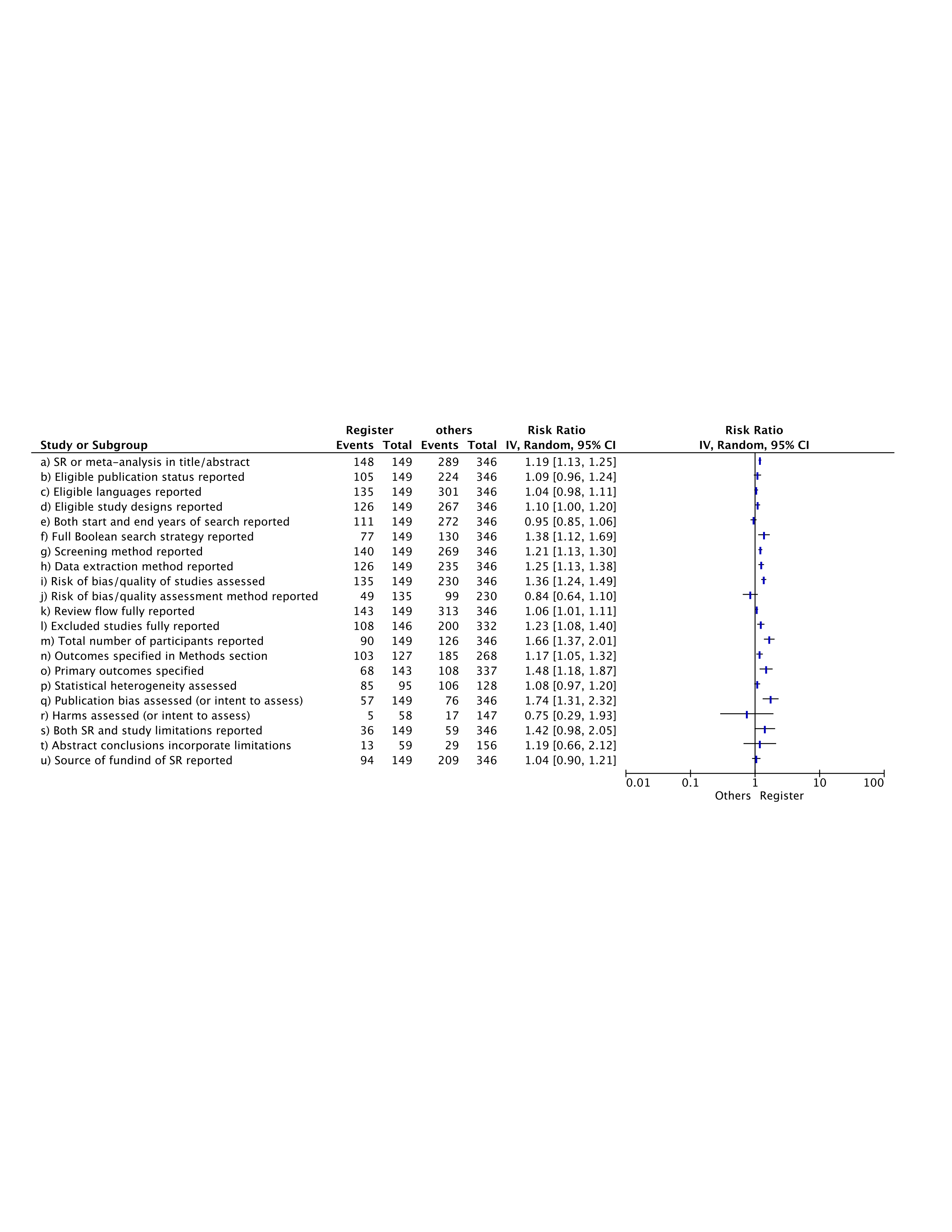

Supplement: Supplementary file 1 — Additional file 1. Study or Subgroup. [file 12874_2020_939_MOESM1_ESM.tiff]
