# Supplementary material for: Protocol registration improves reporting quality of systematic reviews in dentistry
Source: BMC Med Res Methodol. 2020 Mar 11;20:57. doi: 10.1186/s12874-020-00939-7 (PMC7065343; doi:10.1186/s12874-020-00939-7)
Supplement: Supplementary file 2 — Additional file 2. Reporting criteria. [file 12874_2020_939_MOESM2_ESM.docx]

**Reporting criteria**

Domains classified as “not reported” are presented in bold.

1. SR or meta-analysis in title/abstract

Which of the following terms are in the title or abstract of the review?

Systematic review

Meta-analysis

Both "systematic review" and "meta-analysis"

**Neither**

1. Eligible publication status reported

Did the authors make a statement regarding eligibility of studies based on their publication status?

Yes - authors stated both published and unpublished studies were eligible for inclusion (or that no restrictions on publication status were imposed)

Yes - authors stated only published studies were eligible for inclusion

Yes - authors stated only unpublished studies were eligible for inclusion

**No such statement was made**

1. Eligible languages reported

Did the authors make a statement regarding eligibility of studies based on language of publication?

Yes

**No**

1. Eligible study designs reported

Did the authors make a statement regarding eligibility of studies based on their publication status?

Yes - authors stated both published and unpublished studies were eligible for inclusion (or that no restrictions on publication status were imposed)

Yes - authors stated only published studies were eligible for inclusion

Yes - authors stated only unpublished studies were eligible for inclusion

**No such statement was made**

1. Both start and end years of search reported

Were search terms reported for one or more of the electronic databases?

Yes - both start and end dates are reported for ALL databases [Select this option if actual dates are reported, or if reviewers stated that they searched each database "from inception" or placed no limit/restriction on the start date, and reported the date the search was conducted]

Partially - start and end dates are reported for only one of many databases, or only the end date is reported for all databases (e.g. "we searched MEDLINE to May 2013")

**No - not reported for any database**

1. Full Boolean search strategy reported

Were search terms reported for one or more of the electronic databases?

Yes - Full Boolean search logic was reported for one or more database

**Yes - Only main index terms (e.g. MeSH) were reported**

**Yes - Only free text words were reported**

**Yes - Both main index terms and free text words were listed, but no full Boolean search logic was reported**

**No - Readers are referred elsewhere for full search strategy**

**No - No search terms were reported**

1. Screening method reported

What method of study screening did the authors report using?

All titles/abstracts and full text articles were screened by two reviewers independently

All titles/abstracts and full text articles were screened using liberal acceleration

All titles/abstracts and full text articles were screened by one reviewer, and a second reviewer screened a sample of records

All titles/abstracts and full text articles were screened by only one reviewer

Different method applied to titles/abstracts and full text articles

Two reviewers screened records for eligibility, but authors did not specify whether this method was applied independently to both titles/abstracts AND full text articles

**Not reported**

1. Data extraction method reported

What method of data extraction did the authors report using?

Two reviewers independently extracted data from all studies

Two reviewers extracted data from all studies, but authors did not state whether extraction was done independently

One reviewer extracted data from all studies, and another reviewer independently extracted data from a sample of studies

One reviewer extracted data from all studies, and another reviewer checked/verified the extracted data for all (or a sample of) studies

Only one reviewer extracted data from all studies (with no verification by another reviewer)

**Not reported**

1. Risk of bias/quality of studies assessed

Did the authors report assessing the risk of bias (or quality) of the included studies?

Yes

**No**

1. Risk of bias/quality assessment method reported

What method of risk of bias (or quality) assessment did the authors report using?

Two reviewers independently assessed all studies

Two reviewers assessed all studies, but authors did not state whether assessment was done independently

One reviewer assessed all studies, and another reviewer independently assessed a sample of studies

One reviewer assessed all studies, and another reviewer checked/verified the assessments for all (or a sample of) studies

Only one reviewer assessed all studies (with no verification by another reviewer)

**Not reported**

1. Review flow fully reported

Was the review flow (i.e. a description of the number of records screened and included/excluded) reported in the review?

Yes - review flow was reported both in text/table AND in a PRISMA/QUOROM-like flow diagram

Yes - review flow was only reported in a PRISMA/QUOROM-like flow diagram

Yes - review flow was only reported in text/table

**No - review flow was not reported**

1. Excluded studies fully reported

Were the reasons for the exclusion of studies from the systematic review reported?

Yes - reasons for exclusion of ALL excluded full text articles were reported both in text/table AND in a PRISMA/QUOROM-like flow diagram

Yes - reasons for exclusion of ALL excluded full text articles were only reported in a PRISMA/QUOROM-like flow diagram

Yes - reasons for exclusion of ALL excluded full text articles were only reported in text/table

**Partially - reasons for exclusion of only SOME excluded full text articles were reported**

**No - none of the full text articles retrieved were excluded**

**No - reasons for exclusion were not reported for any of the excluded full text articles**

1. **Total number of participants reported**

What was the total number of participants in the systematic review? If this total number is not clearly stated in the report, type 'NR' (e.g. it is not necessary to add up sample sizes reported in a table). Enter either the reported number, 'NR' if not reported, or 'Unclear'. If the unit of analysis is body parts (e.g. eyes, knees), please specify this.

Number

**Not reported**

1. Outcomes specified in Methods section

Did the review authors specify in the Methods section the outcomes that were eligible for inclusion in the review?

Yes

**No**

1. Primary outcomes specified

Did the review authors specify one or more primary outcome(s)?

Yes

**No**

No but only one outcome reported

1. Statistical heterogeneity assessed

Was any method described to formally evaluate statistical heterogeneity of included studies?

Yes

**No**

1. Publication bias assessed (or intent to assess)

Did the authors report assessing (or an intent to assess) publication bias?

Yes, publication bias was assessed

No, publication bias was not assessed, but the authors reported that they intended to assess it if they identified a sufficient number of studies

**No, publication bias was not assessed, and the authors did not report an intention to assess it**

1. Harms assessed (or intent to assess)

Did the authors report an analysis of harms/adverse events (or an intent to analyse harms/adverse events) as part of this systematic review?

Yes - data on harms/adverse events were reported

No - authors reported a plan to analyse harms/adverse events but did not identify any eligible studies, or none of the included studies measured harms/adverse events

**No - authors did not report any harms/adverse events data or any plan to analyse harms/adverse events**

1. Both SR and study limitations reported

Were any limitations reported in the Discussion section? Only answer "Yes" if the authors used terms such as "limitation", "limited" or "flawed" when describing a particular method

Yes – both limitations at the study level (e.g. risk of bias of included studies) and review level (e.g. incomplete retrieval of identified research, reporting bias) were reported

**Yes – only limitations at the study level were reported**

**Yes – only limitations at the review level were reported**

**No limitations were reported**

1. Abstract conclusions incorporate limitations

Was the risk of bias/quality/limitations of the included studies incorporated into the abstract conclusion?

Yes - incorporated

**No – not incorporated (the authors did not acknowledge any limitations throughout the review, or acknowledged some limitations in the Results/Discussion yet ignored this when reporting the abstract conclusion, e.g. state that an intervention works or should be recommended for routine practice, without noting that the evidence on which this conclusion is based was of poor quality)**

1. Source of funding of SR reported

Yes

**No**

**Search strategy**

| **Topic** | **Key-words** |
| --- | --- |
| Dentistry | "Oral Health"[Mesh] OR "Oral Health" OR "Health, Oral" OR "Dentistry"[Mesh]  OR "Dentistry" OR "Dental Research"[Mesh] OR "Dental Research" **AND** |
| Systematic Review | (((systematic review [ti] OR meta-analysis [pt] OR meta-analysis [ti] OR systematic literature review [ti] OR this systematic review [tw] OR pooling project [tw] OR (systematic review [tiab] AND review [pt]) OR meta synthesis [ti] OR meta synthesis [ti] OR integrative review [tw] OR integrative research review [tw] OR rapid review [tw] OR consensus development conference [pt] OR practice guideline [pt] OR drug class reviews [ti] OR cochrane database syst rev [ta] OR acp journal club [ta] OR health technol assess [ta] OR evid rep technol assess summ [ta] OR jbi database system rev implement rep [ta]) OR (clinical guideline [tw] AND management [tw]) OR ((evidence based[ti] OR evidence-based medicine [mh] OR best practice* [ti] OR evidence synthesis [tiab]) AND (review [pt] OR diseases category[mh] OR behavior and behavior mechanisms [mh] OR therapeutics [mh] OR evaluation studies[pt] OR validation studies[pt] OR guideline [pt] OR pmcbook)) OR ((systematic [tw] OR systematically [tw] OR critical [tiab] OR (study selection [tw]) OR (predetermined [tw] OR inclusion [tw] AND criteri* [tw]) OR exclusion criteri* [tw] OR main outcome measures [tw] OR standard of care [tw] OR standards of care [tw]) AND (survey [tiab] OR surveys [tiab] OR overview* [tw] OR review [tiab] OR reviews [tiab] OR search* [tw] OR handsearch [tw] OR analysis [ti] OR critique [tiab] OR appraisal [tw] OR (reduction [tw]AND (risk [mh] OR risk [tw]) AND (death OR recurrence))) AND (literature [tiab] OR articles [tiab] OR publications [tiab] OR publication [tiab] OR bibliography [tiab] OR bibliographies [tiab] OR published [tiab] OR pooled data [tw] OR unpublished [tw] OR citation [tw] OR citations [tw] ,OR database [tiab] OR internet [tiab] OR textbooks [tiab] OR references [tw] OR scales [tw] OR papers [tw] OR datasets [tw] OR trials [tiab] OR meta-analy* [tw] OR (clinical [tiab] AND studies [tiab]) OR treatment outcome [mh] OR treatment outcome [tw] OR pmcbook)) NOT (letter [pt] OR newspaper article [pt]))) |
| Database | PubMed |
| Date limits | from 2017 January 01 until 2017 December 31 |

**
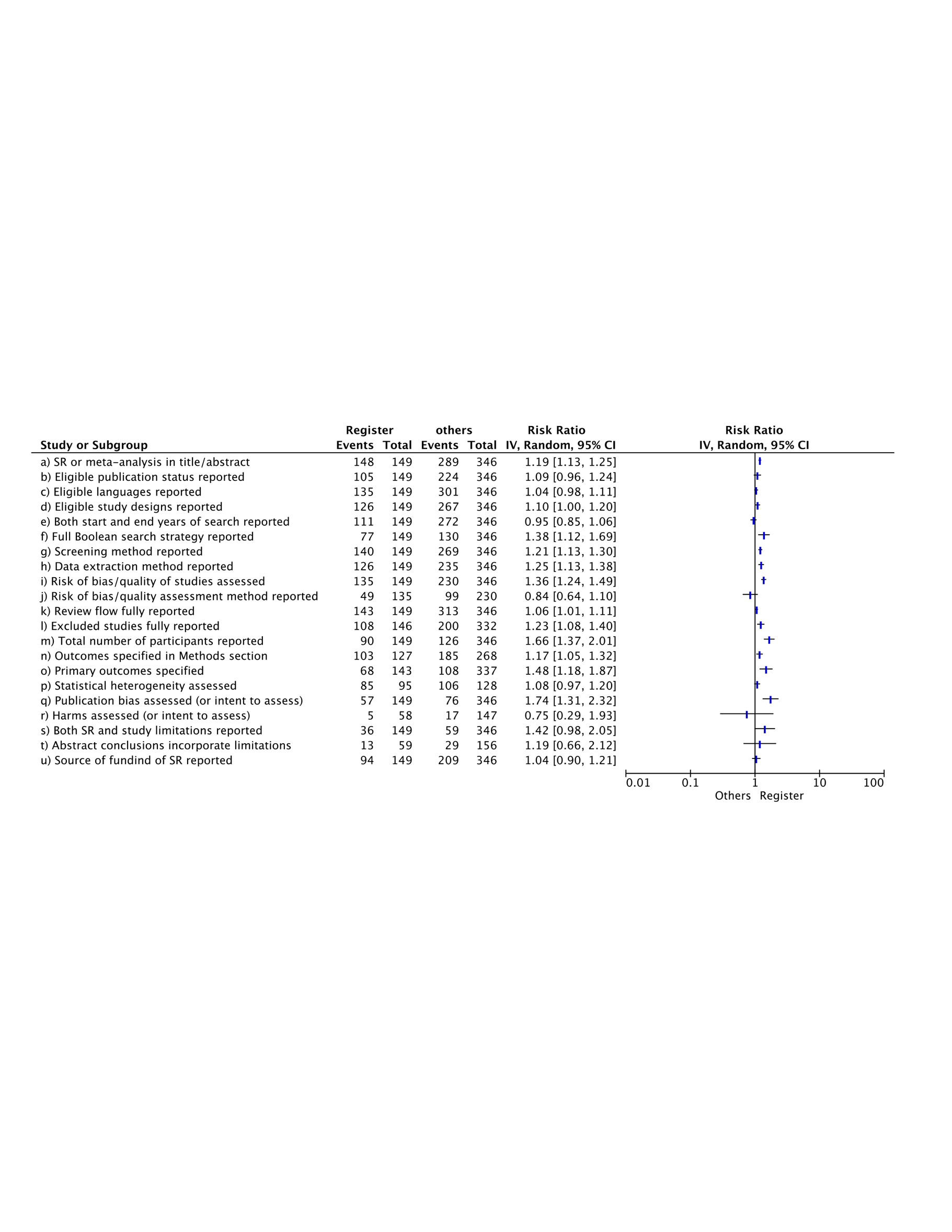
**

Pooled relative risks across assessed reporting characteristics of SRs with 95% confidence intervals comparing the completeness of reporting between SRs that are registered versus the rest.
