# Supplementary material for: Protocol registration improves reporting quality of systematic reviews in dentistry
Source: BMC Med Res Methodol. 2020 Mar 11;20:57. doi: 10.1186/s12874-020-00939-7 (PMC7065343; doi:10.1186/s12874-020-00939-7)
Supplement: Supplementary file 3 — Additional file 3. Rating of each SR considering 21 reporting characteristics. [file 12874_2020_939_MOESM3_ESM.docx]

| Rating of each SR considering 21 reporting characteristics | |
| --- | --- |
| Autor/year | Rating |
| Abduljabbar et al., 2017 | 13/21 |
| Abduljabbar et al., 2017a | 12/21 |
| Afrashtehfar et al., 2017 | 14/21 |
| Agnihotry, et al., 2017 | 15/21 |
| Agnst et al., 2017 | 9/21 |
| Agossa et al., 2017 | 6/21 |
| Ahovuo-Saloranta et al., 2017 | 16/21 |
| Akram et al., 2017 | 15/21 |
| Akram et al., 2017a | 17/21 |
| Akram et al., 2017b | 16/21 |
| Al Makhmari et al., 2017 | 12/21 |
| Al-Akhali and Al-Moraissi, 2017 | 15/21 |
| Al-Hamed et al., 2017 | 11/21 |
| Al-Hamoudi, 2017 | 15/21 |
| Al-Maweri et al., 2017 | 13/21 |
| Al-Moghrabi et al., 2017 | 13/21 |
| Al-Moraissi et al., 2017 | 13/21 |
| Al-Moraissi et al., 2017(a) | 14/21 |
| Al-Thomali et al., 2017 | 10/21 |
| Al-Thomali et al., 2017 | 10/21 |
| Alaizari el al., 2017 | 13/21 |
| Alayyan et al., 2017 | 10/21 |
| Albaker et al., 2017 | 12/21 |
| Aldegheishem et al., 2017 | 7/21 |
| Alharbi et al., 2017 | 3/21 |
| Ali, et al., 2017 | 10/21 |
| Alikhasi et al., 2017 | 12/21 |
| Aljohani et al., 2017 | 6/21 |
| Alkhdra, 2017 | 5/21 |
| AlKudmani et al., 2017 | 13/21 |
| Almangush et al., 2017 | 10/21 |
| Almangush et al., 2017 | 9/21 |
| Almeida et al., 2017 | 15/21 |
| Almeida et al., 2017 | 16/21 |
| Almeida et al., 2017 | 13/21 |
| Almeida et al., 2017 | 9/21 |
| Almeida et al., 2017 | 11/21 |
| Almeida et al., 2017a | 9/21 |
| Altaii et al., 2017 | 9/21 |
| Alves et al., 2017 | 8/21 |
| Amaral et al., 2017 | 6/21 |
| Ambrósio et al., 2017 | 14/21 |
| Aminoshariae et al., 2017 | 10/21 |
| Anand et al., 2017 | 4/21 |
| Antonarakis et al., 2017 | 13/21 |
| Antoszewska-Smith et al., 2017 | 18/21 |
| Armond et al., 2017 | 15/21 |
| Armond et al., 2017 | 15/21 |
| Arora et al., 2017 | 17/21 |
| Arunyanak et al., 2017 | 12/21 |
| Asl et al., 2017 | 5/21 |
| Assaf et al., 2017 | 10/21 |
| Assem et al., 2017 | 14/21 |
| Ata-ali et al., 2017 | 17/21 |
| Ata-ali et al., 2017 | 11/21 |
| Atieh et al., 2017 | 18/21 |
| Atieh et al., 2017 | 14/21 |
| Azaripour et al., 2017 | 15/21 |
| Azarmehr et al., 2017 | 9/21 |
| Azzi et al., 2017 | 8/21 |
| Badran et al., 2017 | 7/21 |
| Baghaie et al., 2017 | 16/21 |
| Baiju et al., 2017 | 8/21 |
| Baiju et al., 2017 | 9/21 |
| Barbosa et al., 2017 | 13/21 |
| Batisse et al., 2017 | 4/21 |
| Benetti et al., 2017 | 13/21 |
| Bertazzo-Silveira et al., 2017 | 12/21 |
| Bertazzo-Silveira et al., 2017 | 12/21 |
| Bertl et al., 2017 | 14/21 |
| Bertl et al., 2017 | 14/21 |
| Bertl et al., 2017 | 14/21 |
| Betti et al., 2018 | 11/21 |
| Bissonntte et al., 2017 | 5/21 |
| Bittencourt et al., 2017 | 12/21 |
| Blázquez-Hinarejos et al., 2017 | 12/21 |
| Bobrowski et al., 2017 | 12/21 |
| Bohner et al., 2017 | 12/21 |
| Borges et al., 2017 | 14/21 |
| Borges Silva et al., 2017 | 15/21 |
| Boronat-Catalá et al., 2017 | 16/21 |
| Bousnaki et al., 2017 | 13/21 |
| Broers et al., 2017 | 14/21 |
| Buj-Acosta et al, 2017 | 9/21 |
| Cai et al., 2017 | 3/21 |
| Cai et al., 2017 | 5/21 |
| Canales et al., 2017 | 11/21 |
| Cardona et al., 2017 | 13/21 |
| Casarin et al., 2017 | 14/21 |
| Casset et al., 2017 | 11/21 |
| Cassina et al., 2017 | 14/21 |
| Castro et al., 2017 | 15/21 |
| Castroflorio et al., 2017 | 12/21 |
| Chau et al., 2017 | 13/21 |
| Chen et al., 2017 | 16/21 |
| Chércoles-Ruiz et al., 2017 | 10/21 |
| Chibinski et al., 2017 | 15/21 |
| Choong Woon & Thiruvenkatachari, 2017 | 16/21 |
| Chrcanovic and Gomez, 2017 | 11/21 |
| Chrcanovic and Gomez, 2017(a) | 10/21 |
| Chrcanovic et al., 2017 | 11/21 |
| Chrcanovic et al., 2017 | 14/21 |
| Chrusciel-Nogalska et al., 2017 | 6/21 |
| Cianetti et al., 2017 | 11/21 |
| Cieplik et al., 2017 | 11/21 |
| Cobein, et al., 2017 | 8/21 |
| Coll et al., 2017 | 16/21 |
| Colombo et al., 2017 | 12/21 |
| Comas-Calonge et al., 2017 | 8/21 |
| Costa et al., 2017 | 17/21 |
| Cotti et al., 2017 | 11/21 |
| Cuba et al., 2017 | 3/21 |
| Cunha et al., 2017 | 15/21 |
| Davoudi, et al., 2017 | 11/21 |
| de Medeiros et al., 2017 | 18/21 |
| Deana et al., 2017 | 16/21 |
| Del Fabbro et al., 2017 | 13/21 |
| Del Monte et al., 2017 | 8/21 |
| Delwel et al., 2017 | 9/21 |
| Deutscher et al., 2017 | 12/21 |
| Divaris et al., 2017 | 9/21 |
| Dogramaci et al., 2017 | 17/21 |
| Dorri et al., 2017 | 18/21 |
| dos Santos et al., 2017 | 10/21 |
| Dougall et al., 2017 | 12/21 |
| Douglas-de-Oliveira et al., 2017 | 18/21 |
| Dourado et al., 2017 | 13/21 |
| Duan et al., 2017 | 14/21 |
| Duangthip et al., 2017 | 9/21 |
| Duggal et al., 2017 | 9/21 |
| Easter et al., 2017 | 17/21 |
| El-Wegoud et al., 2017 | 10/21 |
| Elawady et al., 2017 | 13/21 |
| Elhaddaoui et al., 2017 | 6/21 |
| Elhennawy et al., 2017 | 12/21 |
| Elnayef et al., 2017 | 14/21 |
| Elsaadany et al., 2017 | 10/21 |
| Elshiyab et at., 2017 | 10/21 |
| Esan et al., 2017 | 15/21 |
| Eslami et al., 2017 | 14/21 |
| Eslamipour et al., 2017 | 14/21 |
| Estai et al., 2017 | 11/21 |
| Falci et al., 2017 | 14/21 |
| Feiz et al., 2017 | 8/21 |
| Fernando et al., 2017 | 7/21 |
| Ferreira et al., 2017 | 8/21 |
| Ferreira et al., 2017 | 15/21 |
| Ferreira et al., 2017 | 13/21 |
| Figuero et al., 2017 | 17/21 |
| Finoti et al., 2017 | 9/21 |
| Firmino et al., 2017 | 11/21 |
| Forsyth et al., 2017 | 8/21 |
| Fortuna et al., 2017 | 10/21 |
| Freires et al., 2017 | 11/21 |
| Fueki et al., 2017 | 12/21 |
| Fumes et al., 2017 | 14/21 |
| Gaber et al., 2017 | 11/21 |
| Gaewkhiew et al., 2017 | 12/21 |
| Geisler et al., 2017 | 6/21 |
| Gentile et al., 2017 | 7/21 |
| Geus et al., 2017 | 15/21 |
| Ghaffari et al., 2017 | 11/21 |
| Ghaffari et al., 2017 | 13/21 |
| Ghanem et al., 2017 | 12/21 |
| Ghanem et al., 2017 | 11/21 |
| Gharpure & Bhatavadekar, 2017 | 15/21 |
| Ghasemi et al., 2017 | 3/21 |
| Giudice et al., 2017 | 8/21 |
| Gnanamanickam et al., 2017 | 10/21 |
| Goettems et al., 2017 | 14/21 |
| Goodarzi et al., 2017 | 10/21 |
| Goodwin et al., 2017 | 8/21 |
| Goyak et al., 2017 | 8/21 |
| Guerra et al., 2017 | 7/21 |
| Guimarães et al., 2017 | 10/21 |
| Guzmán-Barrera et al., 2017 | 13/21 |
| Haag et al., 2017 | 9/21 |
| Haas et al., 2017 | 11/21 |
| Hafezeqoran and Koodaryan, 2017 | 11/21 |
| Hamedi-Sangsari et al., 2017 | 12/21 |
| He et al., 2017 | 17/21 |
| He et al., 2017 | 10/21 |
| Heal et al., 2017 | 14/21 |
| Heasman et al., 2017 | 11/21 |
| Helmy et al., 2017 | 16/21 |
| Hendre et al., 2017 | 9/21 |
| Henry et al., 2017 | 15/21 |
| Hettiarachchi et al., 2017 | 9/21 |
| Hindy et al., 2017 | 11/21 |
| Hirsch et al., 2017 | 9/21 |
| Hoben et al., 2017 | 13/21 |
| Hong et al., 2017 | 8/21 |
| Hoogteijling et al., 2017 | 13/21 |
| Hosseinpour et al., 2017 | 4/21 |
| Hsu et al., 2017 | 17/21 |
| Hussein et al., 2017 | 8/21 |
| Iegami et al., 2017 | 8/21 |
| Innes & Schwendicke, 2017 | 15/21 |
| Irving et al., 2017 | 12/21 |
| Isfeld et al., 2017 | 11/21 |
| Islam et al., 2017 | 11/21 |
| Iturriaga et al., 2017 | 10/21 |
| Jacobs et al., 2017 | 7/21 |
| Jafari and Jafari, 2017 | 6/21 |
| Jafari et al., 2017 | 5/21 |
| Jager et al., 2017 | 12/21 |
| Jäger et al., 2017 | 14/21 |
| Jakovljevic et al., 2017 | 10/21 |
| James et al., 2017 | 17/21 |
| Janakiram et al., 2017 | 11/21 |
| Jardim, et al., 2017 | 11/21 |
| Javed et al., 2017 | 12/21 |
| Javed et al., 2017 | 15/21 |
| Javed et al., 2017 | 11/21 |
| Javidi et al., 2017 | 17/21 |
| Jean et al., 2017 | 15/21 |
| Jiménez Garcia et al., 2017 | 11/21 |
| Jiménez-Silva et al., 2017 | 11/21 |
| Jing et al., 2017 | 7/21 |
| Joda et al., 2017 | 6/21 |
| Johson et al., 2017 | 13/21 |
| Jokstad, 2017 | 7/21 |
| Joseph et al., 2017 | 10/21 |
| Juloski et al., 2017 | 3/21 |
| Junior et al., 2017 | 17/21 |
| Kabler et al., 2017 | 4/21 |
| Kalakonda et al., 2017 | 10/21 |
| Kalf-Scholte et al., 2017 | 12/21 |
| Kanzow et al., 2017 | 13/21 |
| Kapferer-Seebacher et al., 2017 | 10/21 |
| Kattadiyil et al., 2017 | 8/21 |
| Kellesarian et al., 2017 | 15/21 |
| Kellesarian et al., 2017 | 15/21 |
| Kellesarian et al., 2017 | 9/21 |
| Kellesarian et al., 2017 | 10/21 |
| Kellesarian et al., 2017 | 14/21 |
| Kellesarian et al., 2017(a) | 12/21 |
| Kellesarian et al., 2017(b) | 10/21 |
| Khadilkar et al., 2017 | 10/21 |
| Khojasteh et al., 2017 | 11/21 |
| Kim et al., 2017 | 7/21 |
| Kinaia et al., 2017 | 13/21 |
| Klasser et al., 2017 | 6/21 |
| Klingberg et al., 2017 | 15/21 |
| Koka et al., 2017 | 6/21 |
| Konstantinidi et al., 2017 | 11/21 |
| Kopp et al., 2017 | 10/21 |
| Kuhn et al., 2017 | 13/21 |
| Kutkut et al., 2017 | 11/21 |
| Lafaurie et al., 2017 | 10/21 |
| Lanau et al., 2017 | 11/21 |
| Leavy et al., 2017 | 11/21 |
| Lee et al., 2017 | 11/21 |
| Lee et al., 2017 | 13/21 |
| Leira et al., 2017 | 14/21 |
| Lemos et al., 2017 | 14/21 |
| Lertpimonchai et al., 2017 | 15/21 |
| Levey et al., 2017 | 11/21 |
| Li et al., 2017 | 12/21 |
| Li et al., 2017 | 12/21 |
| Liébana-Hermoso et al., 2017 | 9/21 |
| Lim et al., 2017 | 15/21 |
| Lima et al., 2017 | 12/21 |
| Lino et al., 2017 | 13/21 |
| Lips et al., 2017 | 12/21 |
| Lizzi et al., 2017 | 7/21 |
| Loffer et al., 2017 | 13/21 |
| Longoni et al., 2017 | 11/21 |
| López-Estudillo et al., 2014 | 11/21 |
| Louro et al., 2017 | 15/21 |
| Lozano-Carrascal et al., 2017 | 11/21 |
| Machado et al., 2017 | 12/21 |
| Mahasneh et al., 2017 | 9/21 |
| Maillard et al., 2017 | 8/21 |
| Mandelaris et al., 2017 | 13/21 |
| Manola et al., 2017 | 5/21 |
| Maran et al., 2017 | 16/21 |
| Marcello-Machado et al., 2017 | 14/21 |
| Marchionatti et al., 2017 | 11/21 |
| Marchionni et al., 2017 | 11/21 |
| Marcussen et al, 2017 | 12/21 |
| Marghalani et al., 2017 | 13/21 |
| Marroquin et al., 2017 | 4/21 |
| Martens et al., 2017 | 12/21 |
| Martinho et al., 2017 | 13/21 |
| Martins et al., 2017 | 13/21 |
| Maske et al., 2017 | 11/21 |
| Mauri-Obradors et al., 2017 | 11/21 |
| McGowan et al., 2017 | 14/21 |
| McGowan, et al., 2017 | 11/21 |
| Mello et al., 2017 | 10/21 |
| Melo et al., 2017 | 12/21 |
| Melo et al., 2017 | 7/21 |
| Mendes et al., 2017 | 14/21 |
| Millet et al., 2017 | 16/21 |
| Millett et al., 2017 | 14/21 |
| Miron et al., 2017 | 7/21 |
| Mishra, et al., 2017 | 5/21 |
| Mizumoto et al., 2017 | 5/21 |
| Mohammed et al., 2017 | 19/21 |
| Mohnish et al., 2017 | 7/21 |
| Monje et al., 2017 | 15/21 |
| Monk et al., 2017 | 17/21 |
| Monsarrat et al., 2017 | 15/21 |
| Moodley et al., 2017 | 3/21 |
| Moraschini et al., 2017 | 17/21 |
| Moraschini et al., 2017 | 17/21 |
| Moreira et al., 2017 | 15/21 |
| Mousoulea et al., 2017 | 15/21 |
| Mozaffari et al., 2017 | 12/21 |
| Mozaffari et al., 2017 | 15/21 |
| Mozynka et al., 2017 | 12/21 |
| Mukherjee et al., 2017 | 8/21 |
| Nagraj S et al., 2017 | 16/21 |
| Nair & Singh, 2017 | 3/21 |
| Nair et al., 2017 | 2/21 |
| Najeeb et al., 2017 | 11/21 |
| Najeeb et al., 2017 | 9/21 |
| Najeeb et al., 2017 | 14/21 |
| Najeeb et al., 2017a | 5/21 |
| Naufel et al., 2017 | 11/21 |
| Naumann et al., 2017 | 8/21 |
| Navarro et al., 2017 | 7/21 |
| Nemezio et al., 2017 | 10/21 |
| Nepomuceno et al., 2017 | 14/21 |
| Noba et al., 2017 | 13/21 |
| Nogueira et al., 2017 | 12/21 |
| Normando et al., 2017 | 16/21 |
| Nosratzehi et al., 2017 | 3/21 |
| Ntovas et al., 2017 | 8/21 |
| O`Rourke et al., 2017 | 6/21 |
| Obadan-Udoh et al., 2017 | 8/21 |
| Oliveira et al., 2017 | 5/21 |
| Oyague et al., 2017 | 6/21 |
| Packiri et al., 2017 | 9/21 |
| Pagotto et al., 2017 | 16/21 |
| Palacios et al., 2017 | 15/21 |
| Papadiochou and Pissiotis, 2017 | 11/21 |
| Papadiochou and Polyzois, 2017 | 12/21 |
| Papageorgiou et al., 2017 | 18/21 |
| Papageorgiou et al., 2017 | 18/21 |
| Papageorgiou et al., 2017 | 14/21 |
| Papageorgiou et al., 2017 | 16/21 |
| Pardal-Peláz & Montero | 6/21 |
| Park et al., 2017 | 10/21 |
| Park et al., 2017 | 10/21 |
| Parra et al., 2017 | 10/21 |
| Patel et al., 2017 | 2/21 |
| Patil et al., 2017 | 7/21 |
| Pauli et al., 2017 | 7/21 |
| Pedrosa et al., 2017 | 10/21 |
| Pelo, et al., 2017 | 9/21 |
| Penoni et al., 2017 | 14/21 |
| Peralta-Mamani et al., 2017 | 12/21 |
| Peraza et al., 2017 | 5/21 |
| Pereira et al., 2017 | 7/21 |
| Person et al, 2017 | 11/21 |
| Pficer et al., 2017 | 18/21 |
| Piekoszewska-Zietek et al., 2017 | 8/21 |
| Pieralli et al., 2017 | 11/21 |
| Plonka et al., 2017 | 5/21 |
| Poggio et al., 2017 | 17/21 |
| Pol et al., 2017 | 12/21 |
| Popli et al., 2017 | 11/21 |
| Poubel et al., 2017 | 11/21 |
| Pourdanesh et al., 2017 | 8/21 |
| Preshaw et al., 2017 | 8/21 |
| Pummer et al., 2017 | 12/21 |
| Quaranta et al., 2017 | 12/21 |
| Rademacher et al., 2017 | 11/21 |
| Rakic et al., 2017 | 17/21 |
| Ramamoorthi et al., 2017 | 13/21 |
| Ramírez et al., 2017 | 9/21 |
| Raoofi et al., 2017 | 5/21 |
| Reda et al., 2017 | 15/21 |
| Reis et al., 2017 | 10/21 |
| Reis et al., 2017 | 12/21 |
| Reissmann et al., 2017 | 12/21 |
| Ribeiro et al., 2017 | 11/21 |
| Riley et al., 2017 | 16/21 |
| Riley et al., 2017 | 18/21 |
| Rivera et al., 2017 | 5/21 |
| Rivera et al., 2017 | 5/21 |
| Romualdo et al., 2017 | 11/21 |
| Rongo et al., 2017 | 12/21 |
| Rosa et al., 2017 | 13/21 |
| Rosa et al., 2017a | 13/21 |
| Rosa et al., 2017b | 9/21 |
| Rossi-Fedele et al., 2017 | 4/21 |
| Rozas et al., 2017 | 12/21 |
| Ruela e+A3:A496t al., 2017 | 14/21 |
| Ruela et al., 2017 | 14/21 |
| Sáez-Alcaide et al., 2017 | 7/21 |
| Salehinejad et al., 2017 | 10/21 |
| Salineiro et al., 2017 | 11/21 |
| Salles et al., 2017 | 11/21 |
| Salzer et al., 2017 | 11/21 |
| Sampaziotiset al., 2017 | 14/21 |
| Sánchez et al., 2017 | 18/21 |
| Santiago et al, 2017 | 15/21 |
| Santinoni et al., 2017 | 11/21 |
| Santos et al., 2017 | 15/21 |
| Savoldi et al., 2017 | 5/21 |
| Schroeder et al., 2017 | 15/21 |
| Schwartz et al., 2017 | 14/21 |
| Schwendicke et al., 2017 | 17/21 |
| Schwendicke et al., 2017 | 9/21 |
| Schwendicke et al., 2017 | 17/21 |
| Sehrawat et al, 2017 | 10/21 |
| Seifi and Matini, 2017 | 6/21 |
| Seminario-Amez et al., 2017 | 5/21 |
| Sendyk et al., 2017 | 16/21 |
| Shah et al., 2017 | 14/21 |
| Shanbhang et al., 2017 | 13/21 |
| Sharma et al, 2017 | 8/21 |
| Siddiqi et al., 2017 | 8/21 |
| Silva et al., 2017 | 13/21 |
| Silva et al., 2017 | 14/21 |
| Silva et al., 2017 | 14/21 |
| Silva et al., 2017 | 16/21 |
| Silva et al., 2017 | 13/21 |
| Siqueira et al., 2017 | 17/21 |
| Sivaramakrishna & Sridharan, 2017 | 17/21 |
| Sivaramakrishnan & Sridharan, 2017 | 15/21 |
| Sivaramakrishnan & Sridhran, 2017 | 10/21 |
| Sivaramakrishnan & Sridhran, 2017 | 16/21 |
| Sivaramakrishnan & Sridhran, 2017 | 14/21 |
| Sivaramakrishnan & Sridhran, 2017 | 14/21 |
| Sket et al., 2017 | 6/21 |
| Skondra et al., 2017 | 9/21 |
| Soares et al., 2017 | 12/21 |
| Soeteman et al., 2017 | 9/21 |
| Sorenson et al., 2017 | 8/21 |
| Soto-Penaloza et al., 2017 | 11/21 |
| Souto-Souza et al., 2017 | 14/21 |
| Stein et al., 2017 | 15/21 |
| Suksaphar et al., 2017 | 9/21 |
| Sun et al., 2017 | 12/21 |
| Suter et al., 2017 | 13/21 |
| Szesz et al., 2017 | 18/21 |
| Taha et al., 2017 | 10/21 |
| Takahashi et al., 2017 | 15/21 |
| Takahashi et al., 2017 | 8/21 |
| Talaván-Serna et al., 2017 | 7/21 |
| Tallarico et al., 2017 | 16/21 |
| Tallarico et al., 2017 | 13/21 |
| Tan et al., 2017 | 9/21 |
| Tan et al., 2017 | 18/21 |
| Tao et al., 2017 | 14/21 |
| Tassi et al., 2017 | 12/21 |
| Tawfik et al., 2017 | 15/21 |
| Teixeira & Corrêa, 2017 | 6/21 |
| Teshome et al., 2017 | 14/21 |
| Thoma et al., 2017 | 12/21 |
| Tong et al., 2017 | 12/21 |
| Toniazzo, et al., 2017 | 16/21 |
| Torabinejad et al., 2017 | 12/21 |
| Torsten et al., 2017 | 6/21 |
| Toti et al, 2017 | 15/21 |
| Travassos et al., 2017 | 16/21 |
| Troconis et al., 2017 | 10/21 |
| Troiano et al., 2017 | 14/21 |
| Troiano et al., 2017 | 16/21 |
| Tsai et al., 2017 | 10/21 |
| Tupyota et al., 2017 | 13/21 |
| Vale et al., 2017 | 14/21 |
| Verweij et al., 2017 | 12/21 |
| Virdee et al, 2018 | 16/21 |
| Virtue et al., 2017 | 11/21 |
| Wang et al., 2017 | 13/21 |
| Wang et al., 2017 | 15/21 |
| Wei et al., 2017 | 13/21 |
| Wessing et al., 2017 | 15/21 |
| Western et al., 2017 | 15/21 |
| Wieckiewicz et al., 2017 | 12/21 |
| Wong et al., 2017 | 12/21 |
| Wu et al., 2017 | 12/21 |
| Wu et al., 2017 | 8/21 |
| Xiang et al., 2017 | 15/21 |
| Xiao et al., 2017 | 11/21 |
| Yang et al., 2017 | 13/21 |
| Yang et al., 2017(a) | 13/21 |
| Yang et al., 2017(b) | 15/21 |
| Yeung et al., 2017 | 10/21 |
| Yin et al., 2017 | 13/21 |
| Yoo et al., 2017 | 13/21 |
| Yusof et al., 2017 | 12/21 |
| Zanolla et al., 2017 | 12/21 |
| Zaror et al., 2017 | 17/21 |
| Zhang et al., 2017 | 10/21 |
| Zhang et al., 2017 | 10/21 |
| Zheng et al., 2017 | 14/21 |
| Zhou et al., 2017 | 10/21 |
| Ziukaite et al., 2017 | 15/21 |
| Zupancic et al., 2017 | 9/21 |
